# Metabolomic richness and fingerprints of deep-sea coral species and populations

**DOI:** 10.1007/s11306-019-1500-y

**Published:** 2019-03-02

**Authors:** Samuel A. Vohsen, Charles R. Fisher, Iliana B. Baums

**Affiliations:** Department of Biology, The Pennsylvania State University, 208 Mueller Laboratory, University Park, PA 16802 USA

**Keywords:** *Callogorgia delta*, Diversity, Rarefaction, Chemotaxonomy

## Abstract

**Introduction:**

From shallow water to the deep sea, corals form the basis of diverse communities with significant ecological and economic value. These communities face many anthropogenic stressors including energy and mineral extraction activities, ocean acidification and rising sea temperatures. Corals and their symbionts produce a diverse assemblage of compounds that may help provide resilience to some of these stressors.

**Objectives:**

We aim to characterize the metabolomic diversity of deep-sea corals in an ecological context by investigating patterns across space and phylogeny.

**Methods:**

We applied untargeted Liquid Chromatography-Mass Spectrometry to examine the metabolomic diversity of the deep-sea coral, *Callogorgia delta*, across three sites in the Northern Gulf of Mexico as well as three other deep-sea corals, *Stichopathes* sp., *Leiopathes glaberrima*, and *Lophelia pertusa*, and a shallow-water species, *Acropora palmata*.

**Results:**

Different coral species exhibited distinct metabolomic fingerprints and differences in metabolomic richness including core ions unique to each species. *C. delta* was generally least diverse while *Lophelia pertusa* was most diverse. *C. delta* from different sites had different metabolomic fingerprints and metabolomic richness at individual and population levels, although no sites exhibited unique core ions. Two core ions unique to *C. delta* were putatively identified as diterpenes and thus may possess a biologically important function.

**Conclusion:**

Deep-sea coral species have distinct metabolomic fingerprints and exhibit high metabolomic diversity at multiple scales which may contribute to their capabilities to respond to both natural and anthropogenic stressors, including climate change.

**Electronic supplementary material:**

The online version of this article (10.1007/s11306-019-1500-y) contains supplementary material, which is available to authorized users.

## Introduction

The three-dimensional structure of colonial cnidarians creates habitat that supports a diverse community of organisms (Öhman and Rajasuriya 1998; Buhl-Mortensen and Mortensen 2005; Idjadi and Edmunds 2006; Roberts et al. 2006). In shallow tropical waters, most corals obtain the bulk of their nutrition from photosynthetic symbionts, creating diverse reef ecosystems that provide an estimated US$ 30 billion annually for the global economy (Cesar et al. 2003). Despite the lack of photosynthesis, deep-sea coral communities similarly support a high diversity of megafauna that includes many commercially important fish species (Jensen and Frederiksen 2011; Costello et al. 2005; Henry and Roberts 2007). Further, the majority of coral species are found in water deeper than 50 meters and deep-sea corals have a very widespread occurrence including continental margins from the Arctic Ocean to Antarctica (Freiwald et al. 1997; Roberts et al. 2006; Post et al. 2010; Yesson et al. 2012). However, deep-sea coral communities are relatively poorly studied due to their inaccessibility.

Corals face a variety of natural and anthropogenic stressors including disease, predation, competition, exposure to oil and other toxins, rising temperatures, and ocean acidification (Guzmán et al. 1991; Slattery et al. 1998; Bruno et al. 2007; Hoegh-Guldberg et al. 2008; García-Matucheski and Muniain 2011; Rasher et al. 2011; White et al. 2012; Perez et al. 2018). The effects of these stressors can range from partial colony mortality to extirpation and ecosystem regime shifts (Bruno et al. 2009; White et al. 2012; Palumbi et al. 2014; Hughes et al. 2017). Being sessile and morphologically simple animals, corals have limited behavioral mechanisms to cope with these stressors. Instead, the diversity of metabolites produced by corals may prove important in their responses to these stressors and to rapid environmental change. Yet, there are only a few detailed investigations of metabolomic profiles in corals.

In plants, the dominant sessile group in terrestrial ecosystems, a diversity of secondary metabolites is involved in herbivore deterrence, communication, competition, and microbial interactions (Weckwerth 2003; Badri et al. 2009; Macel et al. 2010; Holopainen and Blande 2012). Cnidarians are similarly rich in secondary metabolites. For example, terpenes and their derivatives have been the focus of marine products chemists for decades and 90% of known terpenic compounds produced by marine organisms are produced by cnidarians (Kornprobst 2014). Terpenes and their derivatives are involved in many functions in corals, including predator deterrence, anti-fouling and allelopathy, and exhibit medically useful properties such as being antimicrobial, anti-inflammatory and cytotoxic to cancer cell lines (Targett et al. 1983; Sammarco and Coll 1990; Maida et al. 1993; Aceret et al. 1995, 1998; Slattery et al. 1998; Zhang et al. 2005; Chen et al. 2016). Other metabolites have been isolated from corals including caffeine, prostaglandins, natural nitrate esters and alcyopterosins, however the function and properties of many cnidarian compounds remains unknown (Bayer and Weinheimer 1974; Imre et al. 1987; Palermo et al. 2000).

Systematic studies of metabolomic diversity among coral species, populations and individuals may reveal important ecological insights into coral-environment interactions. Analogous exploration of the high genetic diversity found in corals has revealed diverse stress responses between individuals of the same species growing in close proximity (Parkinson et al. 2018). This has led to insights into the scale of environmental variability that is relevant to coral stress responses such as differences observed between populations due to acclimatization and adaptation (Bay and Palumbi 2014; Palumbi et al. 2014). Populations that experience different environmental factors or are genetically divergent are expected to differ in metabolomic composition which may shape their response to stressors. Thus, an understanding of individual and population measures of metabolomic diversity lays the groundwork for exploring the ecological function of the coral metabolome and adds an important phenotyping tool to study the connection between genotype and phenotype.

Here, we applied Liquid Chromatography-Mass Spectrometry targeting lipids to investigate the metabolomic diversity among individuals, populations, and species of corals. We compared three populations of the deep-sea coral *Callogorgia delta* Cairns and Bayer 2002, which is a dominant coral at depths between 400 and 900 m in many hard-bottom communities across the continental slope in the Gulf of Mexico (Quattrini et al. 2013, 2015). Further, *C. delta* was compared to four phylogenetically divergent corals including the globally distributed deep-sea foundation species, *Lophelia pertusa* Linnaeus 1758 (hard coral) and *Leiopathes glaberrima* Esper 1788 (black coral), another deep-sea black coral, *Stichopathes* sp. Brook 1889, and the threatened shallow-water reef-builder, *Acropora palmata* Lamarck 1816 (Lirman 1999; Roark et al. 2006; Cau et al. 2013; Ruiz-Ramos et al. 2015). To the best of our knowledge, this is the first application of high-throughput untargeted metabolomics to deep-sea corals and the first to investigate both individual and population levels of metabolomic diversity of corals across geography and phylogeny.

## Methods

### Sample collection

Five coral species were analyzed for this study: *Acropora palmata, Stichopathes* sp., *Leiopathes glaberrima, Lophelia pertusa* and *Callogorgia delta*. Two *A. palmata* colonies were sampled in the Florida Keys in July 2015: one colony from Sand Island Reef (25.018°N, 80.369°W) and another from French Reef (25.034°N, 80.345°S). Coral fragments were removed using a hammer and chisel and were snap frozen at the surface and stored at − 80 °C until metabolite extraction. Deep-sea corals were collected between April and May of 2015 from the Exploration Vessel Nautilus using a coral cutter mounted on the Remotely Operated Vehicle (ROV) Hercules. Collection locations were named for the Bureau of Ocean Energy Management lease blocks where the sites were located. Three *Stichopathes* sp. colonies were collected from lease block Mississippi Canyon (MC) 344 (1843–1848 m depth, 28.634°N, 88.170°W). Three *Leiopathes glaberrima* colonies and three *Lophelia pertusa* colonies were collected from Viosca Knoll (VK) 906 (394–402 m depth, 29.069°N, 88.378°W). A total of 25 *C. delta* colonies were collected from three sites: MC751 (n = 10, 439–443 m depth, 28.194°N, 89.799°W), MC885 (n = 10, 628–642 m depth, 28.064°N, 89.718°W), and Green Canyon (GC) 234 (n = 5, 509–531 m depth, 27.746°N, 91.223°W). All deep-sea coral samples were placed in a temperature insulated container mounted on the ROV after collection. After recovery of the ROV, samples were placed in cold (< 10 °C) seawater until fragments were subsampled, flash frozen in liquid nitrogen, and then stored at − 80 °C for up to 5 months before metabolite extraction.

### Sample processing

Frozen tissue samples were placed in liquid nitrogen and a fragment of approximately 1 cm in length was subsampled from every colony and individually transferred to 1 mL of extraction solution (0.1% formic acid, 45% isopropanol, 35% acetonitrile, 20% H_2_O, 10 mM Ammonium formate) then homogenized using 5–10 zirconium microbeads at 6500 rpm for 1 min using a PreCellys 24 tissue homogenizer. Samples were then centrifuged at 4 °C for 5 min at 8324 g. Two replicate 250 μL aliquots of supernatant from each extraction were transferred to autosampler vials and stored at − 20 °C for up to two weeks before injection into the mass spectrometer.

Five microliters of each extraction aliquot (n = 72) were injected into an AB Sciex 5600 TripleTOF^®^ mass spectrometer. Lipid ion separation was accomplished using an ACQUITY CSH C18 column (100 mm × 2.1 mm, 1.7 μm particle size) and a gradient elution program with aqueous acetonitrile and isopropanol (10–60%) at a flow rate of 225 µl/min. All samples were run in both positive and negative electrospray ionization modes. Additional details can be found in Online Resource 1. Data were converted from AB Sciex proprietary file formats (.wiff, .wiff.scan, .wiff.mtd) to .mzML in profile mode using MSConvert [ProteoWizard version 3.0.11856] (Chambers et al. 2012; French et al. 2015). Ion peaks were identified and aligned using MS-DIAL (version 2.82) with default parameters: retention time range of 0–100 min, mass range of 0–5000 amu, a Linear Weighted Moving Average smoothing method, smoothing level of 3, minimum peak width of 5, minimum peak height of 3000, retention time tolerance of 0.5 min, and mass tolerance of 0.025 amu (Tsugawa et al. 2015). Putative identities for ions were annotated by comparing fragmentation spectra of samples to database spectra using a retention time tolerance of 0.5 min, MS1 mass tolerance of 0.01 amu, MS2 mass tolerance of 0.05 amu, and an identification score cutoff of 85.

### Data analysis

Samples were internally normalized by total intensity of all putatively identified ions for each sample. Normalized ion intensities and peaks identified by MS-DIAL were exported for statistical analysis in R. Ions detected in positive and negative modes were combined using MSCombine ver1.1 with a mass tolerance of 0.02 amu, time tolerance of 0.5 min, minimum residual of − 0.2 and a maximum residual of 0.2 using all adducts listed by Calderón-Santiago et al. (2016). If a metabolite was detected in both modes, the ion detected in positive mode was retained. To reduce the influence of ion redundancy of diversity measurements, redundant ions resulting from known adducts or isotopologues were removed by custom scripts adapted from the criteria of MS-FLO (DeFelice et al. 2017). In short, redundant ions were identified by mass differences of known adducts, correlations of abundance, and retention times. Further analysis details can be found in Online Resource 1. Species profiles were compared using Principal Component Analysis (PCA) on log_10_ transformed and Pareto-scaled normalized ion intensities. To compare metabolomic richness, ions were considered to be present in a colony only if a peak was detected in both replicates and considered absent only if absent in both replicates. Thus, ions present in only one of two replicates were not considered present nor absent in that colony. Ions were classified as ‘union’ if present in at least one colony of the species or sample group. Ions were classified as ‘core’ if present in all colonies of the species or group. Union and core ions were classified as ‘unique’ to a species or sample group if they were absent in all colonies outside the species or sample group. Ions of *Callogorgia delta* were classified as unique to a site if present at only one site while ignoring other species. Sample groups of higher taxonomic position and habitat [antipatharians (*Leiopathes glaberrima* and *S*. sp.), scleractinians (*Lophelia pertusa* and *A. palmata*), hexacorals (*Leiopathes glaberrima, S*. sp., *Lophelia pertusa*, and *A. palmata*) and deep-sea corals (*Leiopathes glaberrima, S*. sp., *C. delta*, and *Lophelia pertusa*)] were constructed to examine union, core, and unique ions to these groups.

**Fig. 1:**
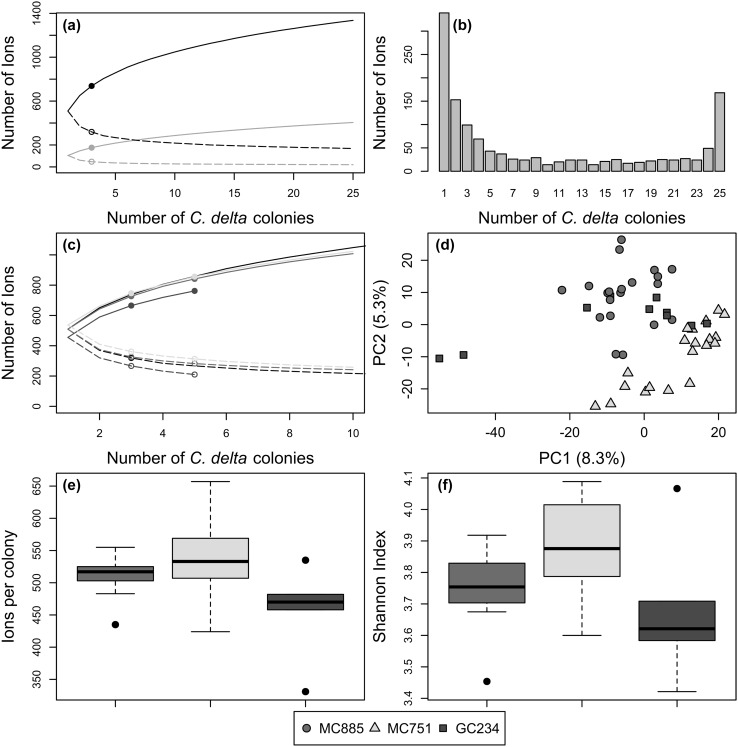
Rarefaction of *Callogorgia delta* ions and differences by site. **a** Rarefaction curves of the union (black), unique union (gray), core (black dashed), and unique core (gray dashed) ions of all *C. delta* colonies. Points show values rarefied to three *C. delta* colonies for comparison to other species. **b** Histogram of the frequency of the detection of each ion in 25 colonies of *C. delta*. **c** Rarefaction curves of the union (solid) and core (dashed) ions of *C. delta* from sites MC885 (blue), MC751 (green), and GC234 (red) and all *C. delta* (black). **d** PCA of metabolomes of *C. delta* colonies from different sites using log_10_ and Pareto-scaled normalized intensities. Two technical replicates for each colony are plotted. **e** Number of ions detected per colony and (f) Shannon indices of *C. delta* from MC885 (blue), MC751 (green), and GC234 (red). Boxes represent first to last quartile while whiskers represent maximum and minimum values excluding outliers. (Color figure online)

Since species and site collections differed in sample size, datasets were rarefied to enable more robust comparisons. For example, to compare *C. delta* (n = 25) to other species (n = 3), the number of core and union ions for every possible combination of three of the 25 colonies (n = 2300 combinations) were calculated. The mean of this distribution was used to compare *C. delta* to other species. Because five colonies were sampled from GC234 and ten each from MC751 and MC885, rarefactions to five colonies were performed on these later sites to compare to GC234 (n = 252 combinations). Rarefaction was also required to compare the number of unique ions across species since ions unique to other species depended on which *C. delta* colonies were subsampled. Thus, the number of unique union and unique core ions were concurrently calculated for all species and all combinations of three of 25 *C. delta* colonies (n = 2300). Further, rarefaction of the union ions unique to each site ignoring other species required concurrent rarefaction of both MC885 and MC751 to five colonies. Each iteration consisted of all colonies from GC234 and five from both MC885 and MC751 (n = 63,504 combinations). The rarefaction curves in Fig. 1 were constructed using 1000 combinations of colonies for each number of colonies in the curve. Wilcoxon rank-sum tests were used to compare Shannon indices and the number of ions per colony between sites and species. No statistical tests were applied to comparisons of population measures of diversity (numbers of union, core, unique union, and unique core ions) due to sample size. Analysis of similarity (ANOSIM) was used to compare the profiles of all species to *Callogorgia delta* using 999 permutations. Replicates from the same colony were combined by averaging the log_10_ and Pareto-scaled normalized intensities for each ion. Clustering analysis was performed using Euclidean distance and Ward’s clustering criterion with 1000 bootstraps.

## Results

### General

A total of 17,182 different ions (10,343 positive, 6839 negative) were detected in all samples. Database matches for 227 ions [151 (1.5%) positive, 76 (1.1%) negative] were identified using MS-DIAL. Positive ion classes detected included acylcarnitines, phosphatidylcholines (PC), lysoPCs, etherPCs, a phosphatidylserine, a sphingomyelin, and triglycerides while negative ion classes detected included lysoPCs, phosphatidylethanolamines (PE), lysoPEs, etherPEs, fatty acids (FA), lyso phosphatidylglycerols, phosphatidylinositols (PI), lysoPIs, and phosphatidylserines. After filtering out potentially redundant ions and combining positive and negative datasets, 7083 ions were retained (6151 positive and 932 negative). Only ions detected in both replicates from at least one colony were considered in further analyses. This included 2753 ions. Seventy-three ions were detected in all colonies of all species.

### Rarefaction curves

The number of union and core ions detected depended strongly on the number of colonies sampled (Fig. 1a). As expected, the number of union and unique union ions increased with additional colonies sampled while the number of core and unique core ions decreased. Further, the union ions did not approach an asymptote by 25 colonies suggesting the metabolomic diversity of the species was not fully sampled. Conversely, the number of core ions approached an asymptote. To understand these patterns, the frequency of occurrence of each ion in the 25 colonies was determined (Fig. 1b). 25% of union ions were present in only one colony. 13% of ions were present in all 25 colonies (core). Similar patterns of increasing number of union ions and decreasing number of core ions with additional colonies sampled were observed when considering each site separately (Fig. 1c).

Population measures of diversity depended strongly on sample size, and thus all further comparisons used rarefaction unless otherwise stated. In addition, since rarefaction of *C. delta* affected the number of unique ions identified in other species and sites, comparisons for all species and sites also used rarefaction unless otherwise stated.

### Site comparison

Principal component analysis showed differences in the metabolomic profiles of *C. delta* by site even though there were no core ions unique to any site (Fig. 1d). The metabolomic profiles of colonies from MC751 and MC885 were distinct and showed complete segregation from each other using the first two components of the PCA (ANOSIM p = 0.001). The distribution of colonies from GC234 overlapped with both MC885 and MC751 in the PCA but their profiles of these colonies were still distinct (ANOSIM p ≤ 0.02). The same patterns held after removal of the apparent outlier colony from GC234. Sites also differed in measures of metabolomic diversity at both the individual and population levels. For most measures, GC234 was the least diverse while MC751 was more diverse (Table 1; Fig. 1e, f). The mean Shannon index for colonies from GC234 was smaller (3.68 ± 0.24) than at sites MC885 (3.75 ± 0.13) and MC751 (3.87 ± 0.16). Similarly, colonies from GC234 had fewer ions per colony (455 ± 75) than those from MC885 (509 ± 32) and significantly fewer than from MC751 (537 ± 61) (two sample Wilcoxon rank-sum test p ≤ 0.043). GC234 had the fewest union (762), core (210), and unique union (30) ions while MC751 had the most union (855), core (312), and unique union (105) ions. There were no core ions unique to any site ignoring other species nor to both site and *C. delta*.

**Table 1:** Comparison of richness and uniqueness of *Callogorgia delta* ions by site

Site	Colonies	Mean ions per colony ± SD	Shannon index^c^	Union ions	Core ions	Rarefied union ions unique to site
GC234	5	455 ± 75	3.68 ± 0.24	762	210	30
MC885	10	509 ± 32	3.75 ± 0.13	842^a^	283^a^	90^b^
MC751	10	537 ± 61	3.87 ± 0.16	855^a^	312^a^	105^b^

Union ions were detected in at least one colony while core ions were detected in all colonies. Sites MC885 and MC751 were rarefied to five colonies each for union and core ions. Ions unique to each site were calculated by concurrently rarefying MC885 and MC751 to five colonies each. Ions were classified as unique to a site if not present in any *C. delta* colony from any other site

^a^Numbers calculated using rarefaction to 5 colonies at MC885 and MC751

^b^Numbers calculated using rarefaction to 5 colonies of both MC885 and MC751, concurrently

^c^Shannon Index was calculated per colony and is displayed as the mean ± standard deviation

### Species comparison

**Fig. 2:**
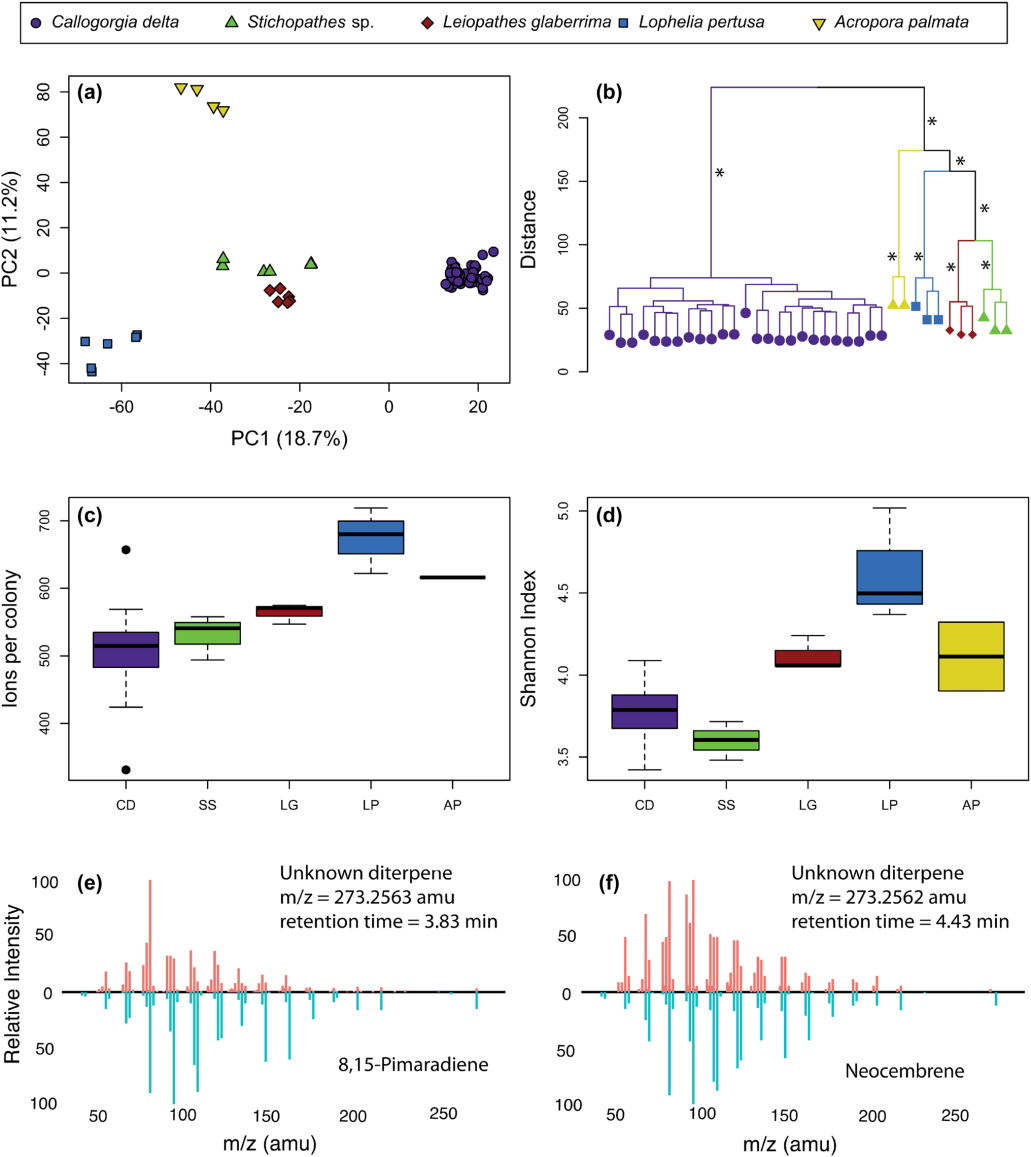
Metabolomic profiles and diversity of five coral species. **a** Principal component analysis (PCA) of log_10_ and Pareto-scaled normalized ion intensities for *Callogorgia delta* (CD, purple circles), *Stichopathes* sp. (SS, green triangles), *Leiopathes glaberrima* (LG, red diamonds), *Lophelia pertusa* (LP, blue squares), and *Acropora palmata* (AP, yellow inverted triangles). Two technical replicates of each colony are plotted. **b** Hierarchical clustering analysis of normalized intensities using combined replicates. * denotes clusters with bootstrap values of 100 after 1000 iterations. **c** Ions detected per colony and **d** Shannon indices for each species. Boxes represent first to last quartile while whiskers represent maximum and minimum values excluding outliers. **e, f** Fragmentation spectra of two core ions unique to *C. delta* samples which match diterpenes such as 8,15-pimaradiene and neocembrene from METLIN. (Color figure online)

The metabolomic profiles of each species were distinct. Principal component analysis demonstrated segregation of all five species using the first two components (Fig. 2a). The degree of segregation among species was greater than among sites for *C. delta. C. delta* was distinct from all other corals (ANOSIM p ≤ 0.002). Several clusters were highly supported that mirrored coral phylogeny and distribution. All species formed their own strongly supported cluster despite small sample sizes. In addition, the two antipatharians, *Leiopathes glaberrima* and *Stichopathes* sp., were clustered together with strong support. Two additional clusters were well supported including all three deep-sea hexacorals and all hexacorals including *A. palmata* (Fig. 2b).

Species differed in individual and population measures of metabolomic diversity. For most measures, *C. delta* was least diverse and *Lophelia pertusa* was most diverse. *C. delta* colonies had the fewest ions per colony (509 ± 60) and lowest Shannon index (3.78 ± 0.18) on average compared to all other species except *Stichopathes* sp. which had a lower Shannon index (Table 2; Fig. 2c, d). *C. delta* had the fewest union ions (738) of all species which ranged from 790 to 1035 ions. Similarly, *C. delta* had 318 core ions which nearly matched *Stichopathes* sp. (317) as the lowest while the other species ranged from 344 to 392 core ions. However, *C. delta* had more unique union ions (175) and unique core ions (48) than both *Stichopathes* sp. (119 and 9) and *Leiopathes glaberrima* (138 and 23).

**Table 2:** Comparison of richness and uniqueness of the metabolomes of coral species

Species	Colonies	Mean ions per colony ± SD	Shannon index^b^	Union ions	Unique union ions	Unique union ions rarefied	Core ions	Unique core ions	Unique core ions rarefied
*Stichopathes* sp.	3	531 ± 33	3.60 ± 0.12	790	85	119^a^	317	6	9^a^
*Leiopathes glaberrima*	3	564 ± 15	4.12 ± 0.11	815	125	138^a^	344	22	23^a^
*Lophelia pertusa*	3	674 ± 49	4.63 ± 0.34	1035	327	362^a^	392	55	63^a^
*Acropora palmata*	2	616 ± 0	4.11 ± 0.30	845	364	383^a^	387	147	152
*Callogorgia delta*	3			738^a^		175^a^	318^a^		48^a^
*C. delta*	25	509 ± 60	3.78 ± 0.18	1336	405		168	20	

Union ions were detected in at least one colony while core ions were detected in all colonies

^a^Numbers calculated using rarefaction of *C. delta* to 3 colonies

^b^Shannon Index was calculated per colony and is displayed as the mean ± standard deviation

*Lophelia pertusa* had the highest Shannon index (4.63 ± 0.34), most ions per colony (674 ± 49), union ions (1035), and core ions (392) by wide margins while *A. palmata* had the most unique union ions (383) and unique core ions (147, Table 2).

### Identified core ions

Twenty-eight ions were identified that were present in all colonies of all species. These included LysoPC 16:0, FA 20:4, ten EtherPCs, and 16 triglycerides which comprised 62% of all triglycerides identified. A total of 13 unique core ions (11 positive, 2 negative) were identified by database matches and were associated with *C. delta, Stichopathes* sp. *Lophelia pertusa, A. palmata*, scleractinians, or deep-sea corals (Table 3). *Lophelia pertusa* had the most with five identified as phosphatidylcholines (three etherPCs and two lysoPCs). Three unique core etherPCs were identified for deep-sea corals, a PC and an etherPC were identified for *A. palmata*, an etherPE was identified for *Stichopathes* sp., and an etherPC and a triglyceride were identified for scleractinians. In addition, two core ions unique to *C. delta* (*m/z* = 273.2563, 273.2562 amu; retention times = 3.84, 4.43 min) were manually annotated as diterpenes based on fragmentation spectrum matches to diterpene hydrocarbons available in the METLIN database including 8,15-pimaradiene and neocembrene (Fig. 2e, f, Smith et al. 2005). The vast majority of unique core ions were not identified by fragmentation spectra. These ions encompassed a wide range of *m/z* and retention times belonging to multiple clusters (Fig. A1c–f, Online Resource 1).

**Table 3:** Putatively Identified core ions to all samples or unique to species or groups

Associated with	Metabolite	Ion	Charge	*m/z* (amu)	Ret. time (min)
*Stichopathes* sp.	EtherPE 33:2e	[M − H]^−^	Negative	686.5184	14.64
*Lophelia pertusa*	LysoPC 22:0	[M + H]^c^	Positive	580.4288	6.19
*Lophelia pertusa*	EtherPC 30:6e	[M + H]^+^	Positive	680.4629	5.07
*Lophelia pertusa*	EtherPC 30:4e	[M + H]^+^	Positive	684.4941	7.43
*Lophelia pertusa*	EtherPC 40:7e	[M + H]^+^	Positive	818.6030	13.45
*Lophelia pertusa*	LysoPC 16:1	[M + FA-H]-	Negative	538.3180	1.95
*Acropora palmata*	PC 27:0	[M + H]^+^	Positive	664.4888	4.54
*Acropora palmata*	EtherPC 38:8e	[M + H]^+^	Positive	788.5562	8.31
*Callogorgia delta*	Unknown diterpene^a^	[M + H]^+^	Positive	273.2563	3.84
*Callogorgia delta*	Unknown diterpene^a^	[M + H]^+^	Positive	273.2562	4.43
Scleractinians	EtherPC 34:3e	[M + H]^+^	Positive	742.5717	12.90
Scleractinians	TG 46:4	[M + NH_4_]^+^	Positive	788.6736	16.98
Deep-sea corals	EtherPC 35:5e	[M + H]^+^	Positive	752.5569	10.35
Deep-sea corals	EtherPC 36:6e	[M + H]^+^	Positive	764.5566	10.24
Deep-sea corals	EtherPC 34:5e	[M + H]^+^	Positive	738.5411	9.03
All samples	LysoPC 16:0	[M + H]^+^	Positive	496.3379	2.51
All samples	EtherPC 34:2e	[M + H]^+^	Positive	744.5877	14.31
All samples	EtherPC 34:1e	[M + H]^+^	Positive	746.6032	14.89
All samples	EtherPC 36:6e	[M + H]^+^	Positive	764.5562	9.29
All samples	EtherPC 36:5e	[M + H]^+^	Positive	766.5723	11.92
All samples	EtherPC 36:4e	[M + H]^+^	Positive	768.5886	14.10
All samples	EtherPC 36:2e	[M + H]^+^	Positive	772.6182	14.95
All samples	EtherPC 37:4e	[M + H]^+^	Positive	782.6017	14.50
All samples	EtherPC 38:4e	[M + H]^+^	Positive	796.6196	14.90
All samples	EtherPC 40:6e	[M + H]^+^	Positive	820.6188	14.48
All samples	EtherPC 40:5e	[M + H]^+^	Positive	822.6357	14.93
All samples	TG 46:2	[M + NH_4_]^+^	Positive	792.7046	17.48
All samples	TG 46:1	[M + NH_4_]^+^	Positive	794.7208	17.74
All samples	TG 47:1	[M + NH_4_]^+^	Positive	808.7375	17.90
All samples	TG 48:3	[M + NH_4_]^+^	Positive	818.7202	17.49
All samples	TG 48:2	[M + NH_4_]^+^	Positive	820.7361	17.76
All samples	TG 48:1	[M + NH_4_]^+^	Positive	822.7523	18.03
All samples	TG 49:1	[M + NH_4_]^+^	Positive	836.7671	18.16
All samples	TG 50:4	[M + NH_4_]^+^	Positive	844.7372	17.57
All samples	TG 50:3	[M + NH_4_]^+^	Positive	846.7517	17.77
All samples	TG 50:2	[M + NH_4_]^+^	Positive	848.7678	18.03
All samples	TG 50:1	[M + NH_4_]^+^	Positive	850.7835	18.28
All samples	TG 52:3	[M + NH_4_]^+^	Positive	874.7830	18.03
All samples	TG 52:2	[M + NH_4_]^+^	Positive	876.7984	18.27
All samples	TG 52:1	[M + NH_4_]^+^	Positive	878.8142	18.51
All samples	TG 54:3	[M + NH_4_]^+^	Positive	902.8140	18.27
All samples	TG 54:2	[M + NH_4_]^+^	Positive	904.8302	18.50
All samples	FA 20:4	[M − H]^−^	Negative	303.2357	4.04

*PE* phosphatidylethanolamine, *PC* phosphatidylcholine, *TG* triglyceride, *FA* fatty acid

^a^Manually annotated

## Discussion

### Sampling the diversity of coral metabolites

*Callogorgia delta* was the most thoroughly sampled species in this study with 25 colonies from three sites. Yet, rarefaction curves for the union ions of all *C. delta* colonies and for each site did not approach an asymptote, suggesting that site and species metabolomic diversity were undersampled (Fig. 1). This high level of metabolomic diversity is also seen in other organisms for which rarefaction curves were applied to LC-MS data (Krug et al. 2008; Bean et al. 2016; Floros et al. 2016). For instance, the diversity detected from a single endophytic fungus did not level off after eighty samples (Maciá-Vicente et al. 2018). The diversity observed in *C. delta* is probably due to the high percentage of union ions detected in only one colony (25%). This is similar to plants where 29% of union ions were in only one of 14 *Arabidopsis thaliana* individuals (Keurentjes et al. 2006). It follows that the diversity of lipids and secondary metabolites of a wide range of organisms, including corals, is high and undersampled.

In contrast, the core ions approached an asymptote indicating that a near complete set of core ions was identified using 25 *C. delta* colonies (Fig. 1). Similarly, the core volatile metabolites detected in *Pseudomonas aeruginosa* cultures leveled off by 24 samples (Bean et al. 2016). In *C. delta*, 13% of union ions were core across all 25 colonies and 15% were core after rarefaction to 14 colonies. This is similar to the 13% shared across 14 *A. thaliana* individuals and 18% among 14 individuals across multiple plant species (Keurentjes et al. 2006; Sawada et al. 2009). Thus, from a population perspective, the majority of ions or metabolites detected in *C. delta* and other organisms are not core. However, at the individual level, a mean of 33% of ions found in a single *C. delta* colony were core to all 25. The discrepancy between population and individual measures of diversity limits the interpretation of diversity patterns. It is certain, however, that the metabolites expressed by individuals of *C. delta* vary considerably, even between individuals growing in close proximity.

Differences in metabolomic diversity between species were detected. Generally, *Callogorgia delta* was the least diverse while *Lophelia pertusa* was most diverse (Table 2; Fig. 2). This held at the individual level with the number of ions per colony and Shannon index as well as at the population level with the number of union and core ions (Table 2; Fig. 2). In other organisms such as bacteria and fungi, differences in richness have been observed between species (Bose et al. 2014; Maciá-Vicente et al. 2018). However, previous work using high-throughput LC-MS on shallow-water corals failed to find differences in diversity between coral species from three scleractinian families using the Shannon index (Quinn et al. 2016). The causes of the diversity differences observed in this study are unclear.

### Chemotaxonomy and function

All coral species investigated had distinct metabolomic profiles (Fig. 2a, b). Similarly, shallow-water corals exhibit distinct species-specific profiles evidenced by multi-dimensional analyses of high-throughput metabolomics data (Sogin et al. 2014, 2017; Farag et al. 2016; Quinn et al. 2016). Moreover, plant and fungal species have also been distinguished with metabolomic profiles (Sawada et al. 2009; Maciá-Vicente et al. 2018). Here, *C. delta* appeared most distinct from the other corals (Fig. 2a, b). This may be due to the fact that *C. delta* is an octocoral which are known to have a distinct secondary chemistry (Kornprobst 2014). The metabolomic profiles of all hexacorals and the two antipatharians also formed distinct groups (Fig. 2b). Thus, metabolomic profiles may be useful to distinguish taxonomic levels above the rank of species in corals.

Unique core ions that may serve as chemotaxonomic markers were detected for all species and phylogenetic groups. Most were unknown while only 13 were putatively identified (Table 3, Fig. A1: Online Resource 1). Additionally, two core ions of *C. delta* were manually identified as diterpene hydrocarbons based on *m/z* and fragmentation spectra. Diterpenes and diterpenoids have been found in many octocorals. They have been shown to be antimicrobial, anti-inflammatory, and toxic to cancer cell lines. Their functions in corals are diverse including predator deterrence, anti-fouling, and allelopathy (Aceret et al. 1995, 1998; Andrianasolo et al. 2007; Chen et al. 2016; Maida et al. 1993; Sammarco and Coll 1990; Slattery et al. 1998; Targett et al. 1983; Zhang et al. 2005). Thus, these diterpenes in *C. delta* may have a medically important function and deep-sea corals may be a source for many more marine natural products. Further, since diterpenes and their derivatives have also been detected using very similar methods in plants, these techniques may be useful to screen for biologically important diterpenes and derivatives in a wide range of organisms (Hu et al. 2005).

Similarly, metabolites specific to individual species and multiple taxonomic groups were discovered for plants and fungi using high-throughput LC-MS (Sawada et al. 2009; Maciá-Vicente et al. 2018). Marine chemists have long known that coral families and genera produce specific secondary metabolites including the family *Primnoidae* to which *C. delta* belongs (see review by Kornprobst 2014). Thus, these unique core ions may have chemotaxonomic utility.

Some unique core metabolites may also be indicators of unique symbiotic partners such as the presence of demospongic acid in *Bebryce studeri* which is derived from its sponge symbiont (Imbs et al. 2009). *Symbiodinium fitti* (ITS2-clade type A3) is an endosymbiont of *Acropora palmata* (Thornhill et al. 2006) which had the most species-specific core ions. Members of the *Symbiodiniaceae* are absent in the other coral species studied here, thus some of *A. palmata*’s unique core ions are likely to be derived from *Symbiodinium*, such as algal-derived fatty acids that are incorporated into membrane lipids. Sogin et al. (2017) showed that the metabolome composition of shallow-water scleractinian corals is correlated with microbial community composition and Imbs et al. (2009) reported that many bacterial fatty acid biomarkers are present in the metabolome of shallow-water coral holobionts. Recent work suggests that shallow-water octocorals have less diverse microbial communities than scleractinians in general (La Rivière et al. 2013, 2015; van de Water et al. 2016, 2017, 2018a, b) and this phylogenetic trend could contribute to the lower metabolomic diversity observed in *C. delta*.

Other unique core ions may be derived from the diet. For example, glaucasterol, a unique algal-derived sterol found in *Acanthogorgia* from over 300 m depth is acquired through a diet of marine snow (Bonini et al. 1983). Marine snow is a substantial dietary component of the four deep-sea corals in our study but not in *Acropora palmata* thus compounds present in marine snow may be detected as core ions unique to the deep-sea corals. Fifteen core ions were unique to deep-sea corals, higher than any other sample group (Table A2, Online Resource 1).

Although no site-specific core ions were found in *Callogorgia delta*, unique union ions, metabolomic profiles, and diversity differed between sites. Shallow-water soft corals also differ in their metabolomic profiles between sites and site differences in richness correlated with water quality in *Nephthea* spp. (Januar et al. 2012; He et al. 2014; Costa-Lotufo et al. 2018). Similarly, metabolomic profiles differ with geography in several plant species (Son et al. 2009; Bernhardsson et al. 2013; Jiang et al. 2014). Here, *C. delta* from sites MC885 and MC751 were most distinct while GC234 was intermediate.

Population genetic patterns in the deep-sea are often structured by depth because depth can be a strong barrier to gene flow (Zardus et al. 2006; Glazier and Etter 2014). In fact, the pattern of metabolomic differentiation in *C. delta* reported here reflects its population genetic structure (Quattrini et al. 2015). Populations of *C. delta* from MC885 and MC751 are genetically distinct from each other while the population from lease block GC235 (adjacent to GC234) is mixed (Quattrini et al. 2015) suggesting that metabolomic divergence in *C. delta* may at least be partially driven by genetics. Genetic divergence may engender metabolomic differences since 75–90% of mass signals obtained by LC-MS were identified as candidates under genetic control in plants (Keurentjes et al. 2006; Gong et al. 2013).

### Conclusion

We used high-throughput metabolomics to examine the diversity of corals from multiple sites and laid the foundation for its use to study the biology and ecology of deep-sea corals. We found that the lipid-targeted metabolome of deep-sea corals is very diverse and composed largely of previously unidentified metabolites. We have shown that species as well as higher taxonomic groups of corals exhibit distinct metabolomic fingerprints and differ in diversity at multiple scales. While these divergent species shared many triglycerides and etherPCs among all colonies, several core ions unique to different taxonomic groups were identified. These are excellent candidates for further study as chemotaxonomic markers or for medically useful properties. Further we have shown that within a species, populations show distinct metabolomic profiles and differ in diversity. Individuals within a population share a small metabolomic core which suggests high metabolic variation between individuals. A better understanding of this metabolomic diversity will help elucidate the diversity of corals’ physiological responses to stressors. Connecting genotype to phenotype is critical to predict the response of these ecologically and economically important organisms to global change.

## Electronic supplementary material

Below is the link to the electronic supplementary material.

Supplementary material 1 (DOCX 98 KB)

Supplementary material 2 (XLSX 20045 KB)

## Data Availability

Analyzed data is available as Online Resource 2. Raw data will be uploaded to Mass Spectrometry Interactive Virtual Environment (MassIVE) (Database ID: MSV000083431).
