# Correction to: Metabolomic richness and fingerprints of deep-sea coral species and populations

**DOI:** 10.1007/s11306-019-1510-9

**Published:** 2019-04-09

**Authors:** Samuel A. Vohsen, Charles R. Fisher, Iliana B. Baums

**Affiliations:** Department of Biology, The Pennsylvania State University, 208 Mueller Laboratory, University Park, PA 16802 USA

## Correction to: Metabolomics (2019) 15:34 10.1007/s11306-019-1500-y

The article “Metabolomic richness and fingerprints of deep-sea coral species and populations”, written by Samuel A. Vohsen, Charles R. Fisher and Iliana B. Baums, was originally published electronically on the publisher’s internet portal (currently SpringerLink) on 02 March, 2019 without open access. With the author(s)’ decision to opt for Open Choice the copyright of the article changed on 30 March, 2019 to © The Author(s) 2019 and the article is forthwith distributed under the terms of the Creative Commons Attribution 4.0 International License (http://creativecommons.org/licenses/by/4.0/), which permits use, duplication, adaptation, distribution and reproduction in any medium or format, as long as you give appropriate credit to the original author(s) and the source, provide a link to the Creative Commons license and indicate if changes were made.

The original article has been corrected.

