# Supplementary material for: Metabolomic richness and fingerprints of deep-sea coral species and populations
Source: Metabolomics. 2019 Mar 2;15(3):34. doi: 10.1007/s11306-019-1500-y (PMC6469635; doi:10.1007/s11306-019-1500-y)
Supplement: Supplementary file 1 — Supplementary material 1 (DOCX 98 KB) [file 11306_2019_1500_MOESM1_ESM.docx]

**Online Resource 1** from:

Metabolomic richness and fingerprints of deep-sea coral species and populations.

**Authors:** Samuel A. Vohsen^1^, Charles R. Fisher^1^, and Iliana B. Baums^1^

**Author Affiliations: ^1^**Department of Biology, The Pennsylvania State University, 208 Mueller Laboratory, University Park, PA 16802, USA

**Corresponding Author:** Samuel A. Vohsen [sav146@psu.edu](mailto:sav146@psu.edu), 636-634-7574

**A1 Additional Chromatography and Mass Spectrometry Settings**

Settings used for chromatography and mass spectrometry followed a standard protocol for lipid analysis by the Metabolomics Facility at the Pennsylvania State University. For each sample, 5µL was separated by reverse phase HPLC using a Prominence 20 UFLCXR system (Shimadzu, Columbia MD) and a Waters CSH C18 column (100mm x 2.1mm, 1.7 um particle size, Milford, MA). The column was held at 55°C and a 20 minute aqueous/acetonitrile/isopropanol gradient elution program using two solvents was used at a flow rate of 225 µL /min. Solvent A was 40% water, 60% acetonitrile with 10mM ammonium formate and 0.1% formic acid, and Solvent B was 90% isopropanol 10% acetonitrile 10mM ammonium formate and 0.1% formic acid. The initial conditions were 60% A and 40 % B, increasing to 43% B at 2 min, 50% B at 2.1 min., 54% B at 12 min, 70% B at 12.1 min and 99% B at 18 min. until 20 min until returning back to initial conditions. The eluate was delivered into a 5600 (QTOF) TripleTOF using a Duospray™ ion source (all AB Sciex, Framingham, MA). The capillary voltage was set at 5.5 kV in positive ion mode and 4.5 kV in negative ion mode, with a declustering potential of 80V. The mass spectrometer was operated in IDA (Information Dependent Acquisition) mode with a 100 ms survey scan from 100 to 1200 m/z, and up to 20 MS/MS product ion scans (100 ms) per duty cycle using a collision energy of 50V with a 20V spread.

**A2 Reducing ion redundancy**

Ion redundancy due to adducts and isotopologues was reduced to limit its influence on measures of diversity such as richness. Ions resulting from the same metabolite were identified and reduced to a single representative ion. A set of criteria to group ions were constructed based on the mass spectral feature list optimizer: MS-FLO (DeFelice et al. 2017). Redundant pairs of ions were identified if their difference in masses matched the mass difference between known ion types. Positive ions investigated included [M+H]+, [M+NH4]+, [M+Na]+, [M+CH3OH]+, [M+K]+, [M+ACN+H]+, [M+2Na-H]+, [M+isopropanol+H]+, [M+ACN+Na]+, [M+2K-H]+, [M+DMSO+H]+, [M+2ACN+H]+, and [M+isopropanol+Na+H]+. Negative ions investigated included [M-H2O-H]-, [M-H]-, [M+Na-2H]-, [M+Cl]-, [M+K-2H]-, [M+FA-H]-, [M+Hac-H]-, [M+Br]-, and [M+TFA-H]-. All combinations of these adducts were screened. A pair of ions was considered redundant if their difference in mass was within 0.02 amu of the difference in mass of known ion types, their difference in retention time did not exceed 0.1 min, and their signal intensities had a correlation greater than 0.75. Signal intensity correlations only utilized samples in which both ions were present. When a pair met all criteria, the ion with the smaller mass was retained unless it was a [M-H20-H]- ion in which case this ion was dropped. For example, the [M-H]- ion would be retained after identifying the pair [M-H]- and [M+Cl-]-. Similarly, the [M+Na]+ ion would be retained after identifying the pair [M+Na]+ and [M+K]+ when no [M+H]+ was detected. This filtering procedure was designed to be conservative by removing all known adducts and isotopologues at the cost of dropping some non-redundant ions

**A3 Ion Identification**

Ions with database matches belonging to the same chemical class clustered together based on mass/charge ratios (m/z) and retention times and separately from other classes (Fig. A1 a-b). For instance, all identified triglycerides belonged to a cluster of positive ions with m/z 700-1000 amu and retention times of 16-19 minutes. In addition, there were distinct clusters of ions without identified members. For example, ions with a wide range of m/z exited the column quickly with short retention times near 1.1 min and are likely polar ions that interacted weakly with the column.

Many of the core ions unique to *A. palmata* and *Lophelia pertusa* fell in the same clusters as those identified as PCs and etherPCs (Fig. A1 c-d). At least one unique core ion of all species and groups fell in a cluster without identified members. Many of the positive and negative unique core ions associated with *Stichopathes* sp. *Leiopathes glaberrima*, *Lophelia pertusa*, *A. palmata*, antipatharians and scleractinians belonged to a cluster with very short retention times and the whole range of m/z where polar compounds are expected.

**A4 Comparisons without rarefaction and with sample groups.**

Some comparisons of population measures of diversity were notable without rarefaction. The number of union and unique union ions for some species and groups exceeded those of others with higher sample sizes. Most notably, *L. pertusa* (n=3) had more union negative ions (1820) and unique union negative ions (581) than *C. delta* (1543 and 388, n=25) despite a far smaller sample size (Table 2). Similarly, the deep-sea corals had more total unique core ions than any other group at 37 despite having the largest sample size (n=34, Table A4).

While rarefaction allowed comparison of the numbers of unique core ions, utilization of the full dataset increased confidence in their uniqueness. Using all samples, unique core ions were still detected for all species and phylogenetic sample groups except negative ions for the hexacorals. A total of 842 (531 positive, 311 negative) unique core ions were identified (Table 2). *A. palmata* had more than any other species alone with 363 (266 positive, 97 negative) followed by *L. pertusa* with 216 (118, 98), *L. glaberrima* with 97 (35, 62), *Stichopathes* sp. with 53 (23, 30), and *C. delta* with 42 (34, 8).


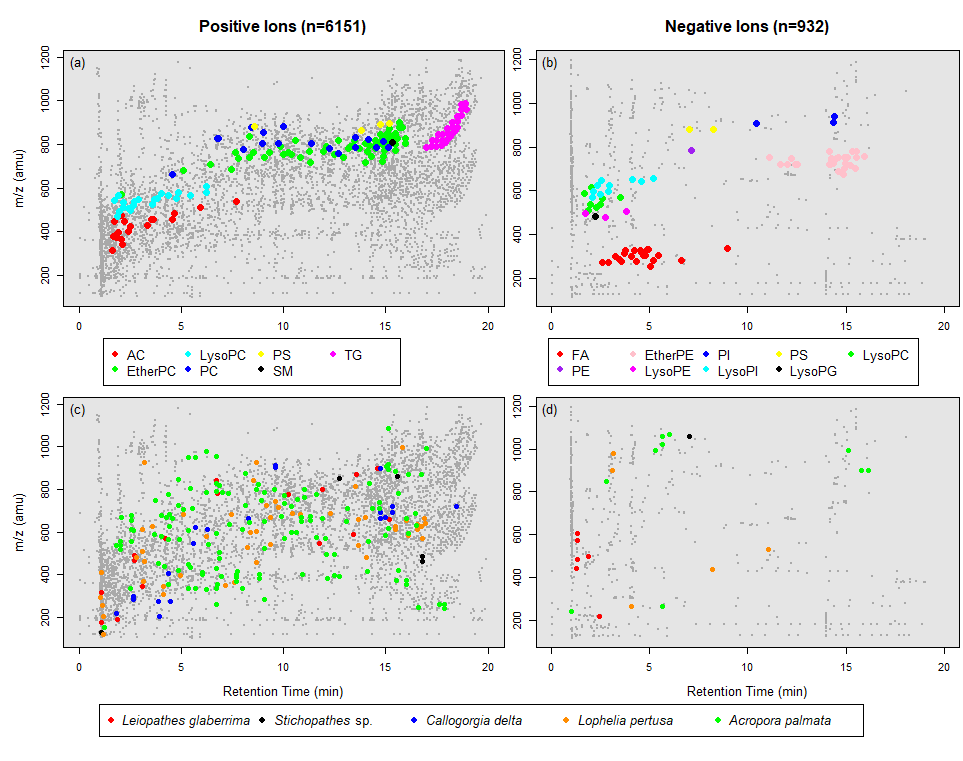


**Fig. A1** Distribution of different groups of ions by mass/charge ratio (m/z in amu) and retention time. All detected ions are displayed in the background of each plot in dark gray. (a-b) Identified ions with database matches by lipid class. AC = acylcarnitine, PC = phosphatidylcholine, PS = phosphatidylserine, SM = sphingomyelin, TG = triglyceride, FA = fatty acid, PE = phosphatidylethanolamine, PI = phosphatidylinositol, PG = phosphatidylglycerol; (c-d) Unique core ions of species. A large cluster of ions have low retention times near 1.1 min and the full range of m/z which includes some of the unique core ions of all species except *C. delta*.

**Table A1** The fragment masses of two diterpenes which match two core ions unique to *Callogorgia delta.* All numbers reported are in amu. The fragment masses of neocembrene and 8-15 pimaradiene were available on the METLIN database and were experimentally acquired using 20eV collision energy in positive mode using a GC APCI Q-TOF mass spectrometer. Fragment masses for the two core ions from *C. delta* with mass/charge ratio (m/z) = 273.2563 amu and retention times 3.84 and 4.42 minutes were acquired in this study.

| **Diterpenes from METLIN** | | **Unique Core ions In *Callogorgia delta*** | |
| --- | --- | --- | --- |
| Neocembrene | 8-15 Pimaradiene | 273.2563_3.84 | 273.2562_4.43 |
| 41.0390 | 41.0393 | NA | NA |
| 43.0553 | 43.0547 | NA | NA |
| 55.0550 | 55.0551 | 55.0580 | NA |
| 57.0711 | 57.0707 | 57.0713 | 57.0732 |
| NA | 65.0395 | 65.0424 | 65.0406 |
| 67.0550 | 67.0548 | 67.0571 | 67.0571 |
| 69.0707 | 69.0705 | 69.0723 | 69.0723 |
| 71.0869 | 71.0853 | 71.0886 | 71.0902 |
| 77.0384 | 77.0395 | 77.0401 | 77.0407 |
| 79.0546 | 79.0549 | 79.0560 | 79.0557 |
| 81.0706 | 81.0705 | 81.0716 | 81.0717 |
| 83.0863 | 83.0858 | 83.0866 | 83.0875 |
| 85.1026 | NA | NA | NA |
| 91.0544 | 91.0546 | 91.0549 | 91.0554 |
| 93.0704 | 93.0704 | 93.0708 | 93.0710 |
| 95.0861 | 95.0860 | 95.0863 | 95.0866 |
| 97.1016 | 97.1016 | 97.1073 | 97.1041 |
| 105.0704 | 105.0702 | 105.0716 | 105.0705 |
| 107.0860 | 107.0859 | 107.0861 | 107.0863 |
| 109.1016 | 109.1014 | 109.1003 | 109.1023 |
| 111.1165 | 111.1170 | NA | 111.1169 |
| 119.0854 | 119.0852 | 119.0857 | 119.0859 |
| 121.1016 | 121.1015 | 121.1023 | 121.1034 |
| 123.1170 | 123.1173 | 123.1149 | 123.1168 |
| NA | 125.1331 | NA | 125.1366 |
| 133.1014 | 133.1010 | 133.1002 | 133.1010 |
| 135.1170 | 135.1174 | 135.1162 | 135.1166 |
| 137.1330 | 137.1326 | 137.1306 | 137.1310 |
| 147.1167 | 147.1172 | 147.1162 | 147.1167 |
| 149.1326 | 149.1329 | 149.1328 | 149.1332 |
| 151.1490 | 151.1468 | NA | NA |
| 161.1328 | 161.1333 | 161.1298 | 161.1321 |
| 163.1482 | 163.1487 | 163.1423 | 163.1479 |
| 165.1648 | 165.1654 | 165.1589 | 165.0691 |
| 175.1484 | 175.1482 | 175.1492 | 175.1462 |
| 177.1637 | 177.1642 | 177.1663 | NA |
| 189.1644 | 189.1650 | 189.1598 | NA |
| 191.1800 | 191.1803 | 191.1795 | NA |
| 203.1796 | 203.1804 | 203.1772 | NA |
| 217.1956 | 217.1963 | 217.1863 | NA |
| 231.2118 | 231.2119 | NA | NA |
| 258.2327 | 258.2299 | NA | NA |
| 273.2583 | 273.2591 | 273.2554 | NA |

**Table A2** Comparison of richness and uniqueness of groups of coral species. Union ions were detected in at least one colony while core ions were detected in all colonies.

| **Group** | **Colonies** | **Union ions** | **Unique union ions** | **Core Ions** | **Unique Core Ions** |
| --- | --- | --- | --- | --- | --- |
| Antipatharians | 6 | 1153 | 247 | 218 | 4 |
| Scleractinians | 5 | 1596 | 742 | 127 | 5 |
| Hexacorals | 11 | 2147 | 1225 | 95 | 2 |
| Deep-sea corals | 34 | 2331 | 1763 | 91 | 15 |
| All | 36 | 2753 |  | 73 |  |
